# Identification of Six Genes as Diagnostic Markers for Colorectal Cancer Detection by Integrating Multiple Expression Profiles

**DOI:** 10.1155/2022/3850674

**Published:** 2022-07-22

**Authors:** Peijie Wu, Xiuqing Yang, Ling Qiao, Yanju Gong

**Affiliations:** ^1^School of Basic Medical Sciences, Chengdu University of Traditional Chinese Medicine, Chengdu 611130, China; ^2^Department of Pharmacy, Chengdu Women's and Children's Central Hospital, School of Medicine, University of Electronic Science and Technology of China, Chengdu 610041, China

## Abstract

**Background:**

Many studies have demonstrated the promising utility of DNA methylation and miRNA as biomarkers for colorectal cancer (CRC) early detection. However, mRNA is rarely reported. This study aimed to identify novel fecal-based mRNA signatures.

**Methods:**

The differentially expressed genes (DEGs) were first determined between CRCs and matched normal samples by integrating multiple datasets. Then, Least Absolute Shrinkage and Selection Operator (LASSO) regression was used to reduce the number of candidates of aberrantly expressed genes. Next, the potential functions were investigated for the candidate signatures and their ability to detect CRC and pan-cancers was comprehensively evaluated.

**Results:**

We identified 1841 common DEGs in two independent datasets. Functional enrichment analysis revealed they were mainly related to extracellular structure, biosynthesis, and cell adhesion. The CRC classifier was established based on six genes screened by LASSO regression. Sensitivity, specificity, and area under the ROC curve (AUC) for CRC detection were 79.30%, 80.40%, and 0.85 (0.76–0.92) in the training set, and these indexes achieved 93.20%, 41.80%, and 0.73 (0.65–0.83) in the testing set. For validation set, the sensitivity, specificity, and AUC were 98.90%, 98.00%, and 0.97 (0.94–0.99). The average sensitivities exceeded 90.00% for CRCs with different clinical features. For adenomas detection, the sensitivity and specificity were 74.50% and 64.00%. Besides, the six genes obtained an average AUC of 0.855 for pan-cancer detection.

**Conclusion:**

The six-gene signatures showed ability to detect CRC and pan-cancer samples, which could be served as potential diagnostic markers.

## 1. Introduction

Colorectal cancer (CRC) is a collection of neoplastic diseases for colon cancer and rectal cancer, which occur in the colon and rectum, respectively. CRC is one of the top 5 most common cancers in both men and women worldwide [1], and in China, it has seen a rapidly increased burden with the change of lifestyle behaviors and improved dietary [2]. The high heterogeneity of CRC has been reported in recent years due to the identified subgroups which showed different prognosis and response to therapies according to distinctive clinical or molecular features [3–5]. Many risk factors have been associated with CRC, such as unbalanced diet, alcohol abuse, smoking, and obesity [6, 7]. The general population in many regions will develop CRC with 5% lifetime risk, and approximately 45% of CRCs die due to the disease [8]. The average 5-year overall survival rate of CRC is about 60%∼70% for all diagnosed patients, while a worse overall survival rate is observed for patients with elder age and late stage [9, 10].

Given the slow development of CRC from precursor lesions, patients used to be diagnosed in the advanced stages when they are aware of it, resulting in a poor prognosis. Appropriate screening and early detection of CRC will facilitate mitigating the incidence and mortality of the disease [11]. Genomic variation-based assays showed great assistance in precision medicine and target therapies of CRC; however, the high heterogeneity of DNA mutations among CRCs makes them not the optimal biomarker candidates [12]. Colonoscopy and guaiac fecal occult blood test (gFOBT)/fecal immunochemical test for hemoglobin (FIT) are the three predominant screening tools used currently for the screening of CRC. Colonoscopy has been recognized as the gold standard, while the disadvantages are mainly poor patient compliance and being invasive [13]. gFOBT/FIT are two fecal-based noninvasive tests that are easy to be performed at home, whereas false-negative results could be produced due to sporadic bleeding, and not all lesions in early stage bleed frequently [14].

Molecular marker assays present several practical advantages when comparing with the current widely used methods [15] and a series of prognostic signatures are identified for cancers [16–19]. In particular, noninvasive tests such as fecal-based biomarkers provide a chance for early CRC diagnosis. Based on the significant exfoliation of dysplastic cells from colorectal lesions into the lumen, host mRNA in the stools has the great potential to be a biomarker [20]. It has been demonstrated that target mRNAs originated from the tumor or surrounding tissues and that expression is not affected by the primary location but is associated with the tumor size and transcript expression level in the tumor [21]. Therefore, fecal-based mRNA detection is suitable and reliable to serve as a biomarker for CRC diagnosis and prognosis prediction. Several stool-based mRNAs such as cyclo-oxygenase 2 (*COX*-2) [22] or matrix metalloproteinase 7 (*MMP*-7) [23] were also reported that have the ability to discriminate CRC from normal controls. Nevertheless, fecal-based mRNA biomarkers have not been comprehensively explored in CRC. Compared with single mRNA biomarker, a gene signature containing multiple mRNA biomarkers are more accurate.

Therefore, in this study, we aim to identify a novel fecal-based mRNA signature that could act as a new noninvasive approach for CRC detection. For this purpose, the differentially expressed genes (DEGs) were first determined between CRCs and matched normal samples by integrating multiple datasets retrieved from the GEO database. The DEGs were then screened via Least Absolute Shrinkage and Selection Operator (LASSO) regression to reduce the number of candidates of aberrantly expressed genes. We further developed a CRC classifier using the logistic regression model based on the screened candidates. The performance of the classifier for the detection of CRC and precancerous adenomas was finally comprehensively evaluated using the expression profiles of stool samples and CRC tissue samples. We believe the constructed classifier provides a possible strategy that could be used for CRC detection and screening.

## 2. Materials and Methods

### 2.1. Data Preparation and Preprocessing

Five eligible microarray datasets of colorectal cancer were collected from the Gene Expression Omnibus (GEO, https://www.ncbi.nlm.nih.gov/geo/) database, of which four datasets were generated by Affymetrix platform (GSE106582, GSE41258 [24], GSE44076 [25],and GSE99573 [26]) and one was generated from Illumina HumanHT-12 platform (GSE117606). The criteria for selecting GEO datasets are as follows: each consists of at least ten tumor and paired normal samples, and all are generated by the same platform. For stool samples, at least 100 normal and tumor samples are required. To reduce the batch effect among different datasets, we retrieved the raw “CEL” files of the Affymetrix platform. Affy *R* package (https://www.bioconductor.org/packages/release/bioc/html/affy.html) was used to process CEL files and then obtain the expression profiles of probes. The expression values were then normalized by the robust multiarray averaging (RMA) method [27]. Patient clinical features were also retrieved if available, and a total of 1126 samples were finally used in this study (Table 1).

### 2.2. Differential Expression Analysis

Differentially expressed genes (DEGs) between tumor and matched normal samples were then determined using limma *R* package [28] in GSE106582 and GSE117606 datasets for both of them were generated by the Affymetrix® platform. DEGs were screened under the false discovery rate (FDR) <0.05.

### 2.3. Functional Enrichment Analysis

Gene Ontology (GO) enrichment and Kyoto Encyclopedia of Genes and Genomes (KEGG) pathway analysis were performed using the *R* package of clusterProfiler (version 3.8) [29]. The significantly enriched GO terms and pathways were identified with q value less than 0.05. Top 15 enriched GO and KEGG terms were showed using the “dotplot” method in clusterProfiler package. Gene set expression analysis was implemented in GSEA software (v3.0) [30] with default parameters (permutation = 1000). The c2.cp KEGG pathway gene set (v6.2) was selected. KEGG pathways with FDR less than 0.05 were deemed significant.

### 2.4. Developing CRC Classification Model

The GSE99573 dataset was used to develop a CRC classification model because the samples were collected from feces, which is more compliant for individuals. The LASSO (Least Absolute Shrinkage and Selection Operator) Cox regression was used for feature selection. Briefly, LASSO regression can result in sparse models with few coefficients by performing L1 regularization, which is ideal for producing simpler models. Some coefficients can be set equal to zero and eliminated from the model when larger penalties are selected. Ten-fold cross-validation was conducted to determine the best lambda and avoid overfitting. The measured type was set “deviance,” also known as −2log-likelihood, for the cross-validation loss. The CRC classification model was defined as Σ (*βi* ^*∗*^Expi), where *β* indicates Cox coefficients, Exp indicates the expression level, and *i* indicates genes. Firstly, the normal and CRC samples are randomly divided into a training set and testing set equally (seed = 54213). The classification model was then developed using logistic regression based on the training set and verified in the testing set.

### 2.5. Validation of the Classification Model

GSE44076 and GSE41258 were used as the external validation set because both are generated from the Affymetrix platform, and both are CRC tissue samples. The downloaded raw data were first normalized by the RMA method before they were merged into one dataset. Batch effects between the datasets were removed using the “removeBatchEffect” method in the limma *R* package [28]. The merged dataset contained 486 samples, with 202 and 284 normal and tumor samples, respectively.

### 2.6. Evaluation the Performance for Pan-Cancer Classification

Gene expression profiles of multiple cancer types were downloaded from the TCGA pan-cancer project (https://api.gdc.cancer.gov/data/3586c0da-64d0-4b74-a449-5ff4d9136611). The expression profiles of 14 cancers were further retrieved, including esophageal carcinoma (ESCA), stomach adenocarcinoma (STAD), colorectal cancer (CRC), liver hepatocellular carcinoma (LIHC), pancreatic adenocarcinoma (PAAD), ovarian serous cystadenocarcinoma (OV), uterine corpus endometrial carcinoma (UCEC), cervical and endocervical cancers (CESC), breast invasive carcinoma (BRCA), lung adenocarcinoma (LUAD), lung squamous cell carcinoma (LUSC), head and neck squamous cell carcinoma (HNSC), urothelial bladder carcinoma (BLCA), and prostate adenocarcinoma (PRAD). Only the primary cancer samples were included, and 6432 samples were obtained (Supplementary Table 1). The matched normal samples of the 14 cancer types were defined as a normal group, including 514 samples.

### 2.7. ROC Curve Analysis

The ROC curve analysis was implemented using the *R* package “pROC” [31]. The predicted probability of each sample was estimated by the logistic regression model and is used as a predictor. The normal and cancer samples were assigned 0 and 1, respectively, and used as a response variable. The area under the ROC curve (AUC) was then calculated. Finally, the optimal cutoff and the corresponding sensitivity and specificity were determined using the maximized Youden index.

### 2.8. Statistical Analysis

Statistical analyses and figure plotting were performed using *R* (version 3.6.1) software. Rtsne and prcomp methods were used to conduct PCA and *t*-SNE analysis. The two methods allowed us to obtain a small number of principal components by reducing the large number of variables, also known as dimension reduction. The top 2 principal components were then used to show the dataset structure in two-dimensional coordinates. Statistical significance for the comparisons of paired and unpaired two groups was estimated by paired Student's *t*-test and Mann–Whitney *U* test, respectively. Kruskal–Wallis test was used for the comparisons of more than two groups. *P* values were adjusted by Benjamini–Hochberg method, which was also called FDR. In this study, we assigned the symbols of “^*∗∗∗*^,” “^*∗∗*^,” and “^*∗*^” representing *P* < 0.001, *P* < 0.01, and *P* < 0.05, respectively.

## 3. Results

### 3.1. Overall Design of This Study

**Figure 1:**
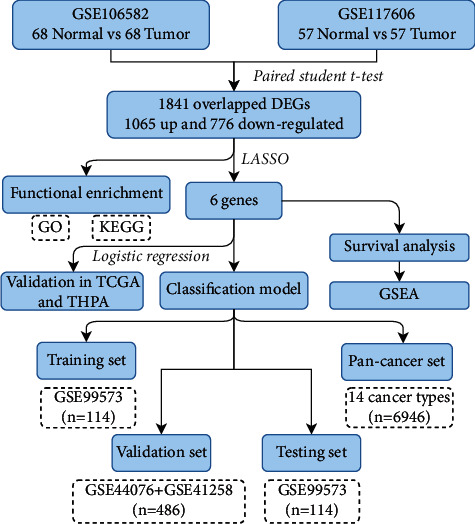
Flowchart of this study. DEGs: different expression genes. GO: Gene Ontology. KEGG: Kyoto Encyclopedia of Genes and Genomes. LASSO: Least Absolute Shrinkage and Selection Operator. TCGA: The Cancer Genome Atlas. THPA: The Human Protein Atlas. GSEA: gene set enrichment analysis.

The design of this study is showed in Figure 1. Firstly, the differentially expressed genes (DEGs) between tumor and matched normal samples were identified using the expression profiles of GSE106582 and GSE117606 datasets separately by performing paired student's *t*-test. The overlapped DEGs of the two datasets were selected for following analysis. Enrichment analysis of Gene Ontology (GO) and the KEGG pathway was conducted to investigate the potential functions of the filtered DEGs. By using the LASSO regression method and stool samples provided by the GSE99573 dataset (Table 2), we identified six candidate genes from 1841 overlapped DEGs and further developed a logistic regression model for the classification of CRCs and pan-cancer samples, which herein was designated as the 6-gene classification model. The relationship between the six genes and CRC prognosis was also investigated by using TCGA CRC dataset.

### 3.2. Identification of DEGs

**Figure 2:**
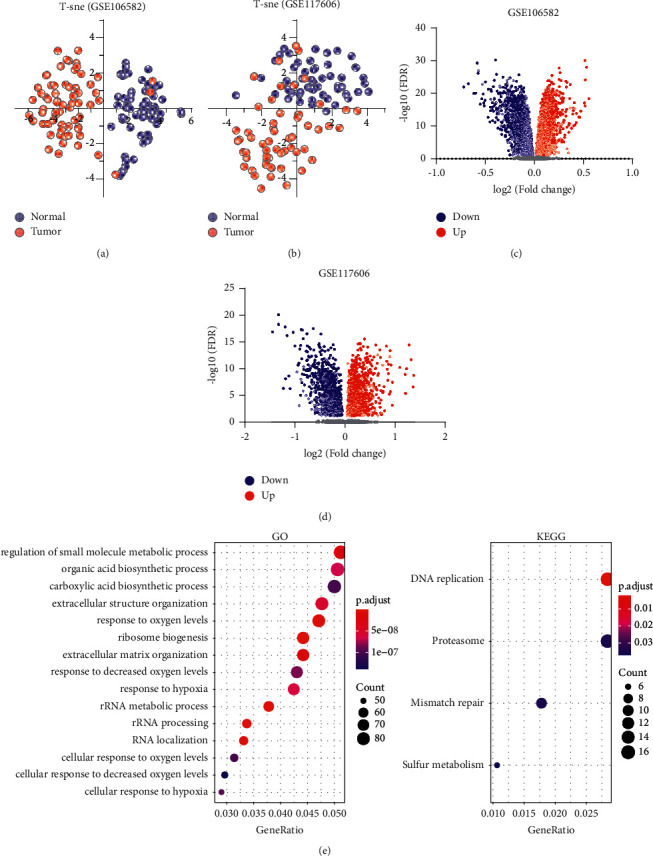
Identification of differentially expressed genes. (a)-(b): *t*-SNE plots of the tumor and normal samples on GSE106582 (a) and GSE117606 (b) datasets, respectively. The blue dots and red dots represent normal and tumor samples. (c)-(d): Volcano plots show the fold change and FDR of differentially expressed genes identified by GSE106582 (c) and GSE117606 (d) datasets. (e) GO and KEGG enrichment of the overlapped DEGs. Top 15 enriched GO terms are presented.

The similarity between the tumor and normal samples in each dataset was first estimated by the *t*-distributed stochastic neighbor embedding (*t*-SNE) method using all gene expressions. Tumor and normal samples in both datasets were separated from each other clearly and clustered together in each group, indicating a high consistency of the two groups (Figures 2(a) and 2(b)). Differential expression analysis identified 6285 (up/down: 3424/2861) and 2408 (up/down: 1367/1041) DEGs in the GSE106582 and GSE117606 datasets, respectively (Figures 2(c) and 2(d)). The overlapped DEGs were defined as that the genes in both datasets were up- or downregulated. A total of 1841 DEGs were obtained (up/down: 1065/776, Supplementary Table 1), which accounted for 29.29% and 76.45% of the whole DEGs identified in GSE106582 and GSE117606 datasets. GO enrichment analysis revealed that the overlapped DEGs were mainly associated with the biological processes of small molecule metabolism, extracellular structure, and biosynthesis (Figure 2(e), Supplementary Table 2). Pathway analysis indicated that these genes were significantly enriched in DNA replication, proteasome, mismatch repair, and sulfur metabolism (Supplementary Table 3).

### 3.3. Identification of the Six-Gene Signature

**Figure 3:**
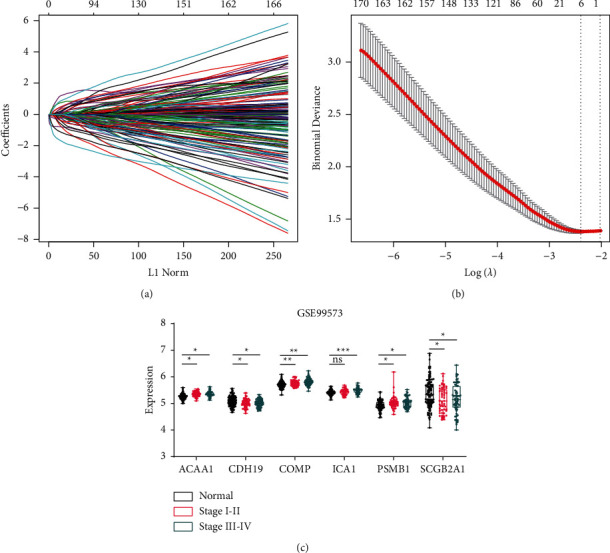
Six genes identified by LASSO regression. (a) The path of the coefficients against the ℓ1-norm of the whole coefficient vector as *λ* varies. Each curve represents the track of a variable. The axis above indicates the number of nonzero coefficients at the current *λ*. (b) The deviances against different *λ*. Error bars show the 95% confidence intervals of each deviance. The left and right dashed lines indicate the minimized *λ* (lambda.min) and the 1se lambda, respectively. (c) The expression profiles of the six genes between normal and CRCs which were collected from stool samples. All CRC samples were divided to early stage (I-II) and late stage (III-IV) according to the patient stage.

The LASSO regression was used to produce a simple model with fewer variables by eliminating some redundant or multicollinearity parameters. The tuning parameter *λ* was adjusted during the LASSO procedures to control the strength of the L1 penalty. We found that the deviance essentially reached a minimum when lambda equaled 0.091, where the corresponding number of variables was 6 (Figures 3(a) and 3(b)). Further analysis indicated that the six genes were differentially expressed between normal and tumor samples, with *ACAA1* and *PSMB1* being upregulated and *CDH19*, *COMP*, *ICA1*, and *SCGB2A1* downregulated in tumor samples (Figure 3(c)).

**Figure 4:**
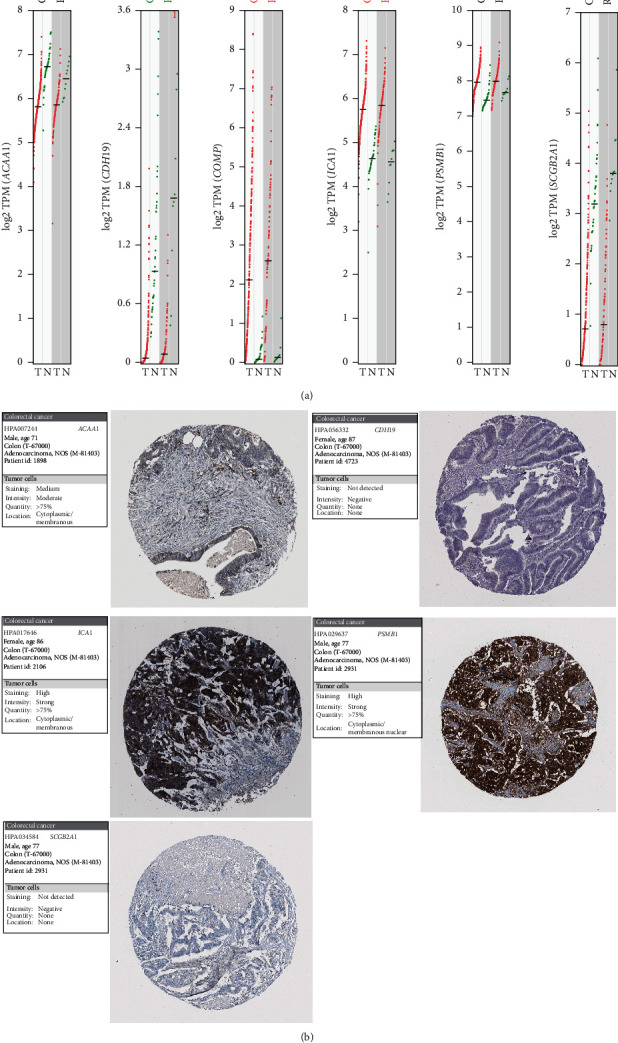
Validation of the six genes in TCGA CRC dataset and THPA database. (a) Expression profiles of the six genes in TCGA CRCs. COAD: colon adenocarcinoma. READ: rectum adenocarcinoma. The red and green dots indicate the expression values of tumor and normal samples, respectively. Significantly upregulated genes in tumor or normal samples are displayed by the red or green text in the top. (b) Images of immunohistochemistry stained CRC tissues. The examples of five genes were obtained from The Human Protein Atlas.

In the TCGA CRC dataset, three genes, *ACAA1*, *CDH19,* and *SCGB2A1,* were downregulated in tumor tissues, whereas the other three genes were overexpressed in tumor tissues (Figure 4(a)). To verify the expression change in CRC, we obtained the immunohistochemistry data of five genes (*ACAA1*, *PSMB1*, *CDH19*, *ICA1*, and *SCGB2A1*) from The Human Protein Atla database (*COMP* is not included as no data could be obtained) [32]. We found that *CDH19* and *SCBB2A1* displayed negative signal intensity in the IHC stained cancer tissues, while *ACAA1* showed moderate intensity in cancer tissues. The other two genes, *ICA1* and *PSMB1*, were observed displaying very strong intensity in cancer tissues (Figure 4(b)). These results demonstrated a good agreement between the gene expression levels and protein expression levels.

### 3.4. Prognosis Analysis of the Six Genes

**Figure 5:**
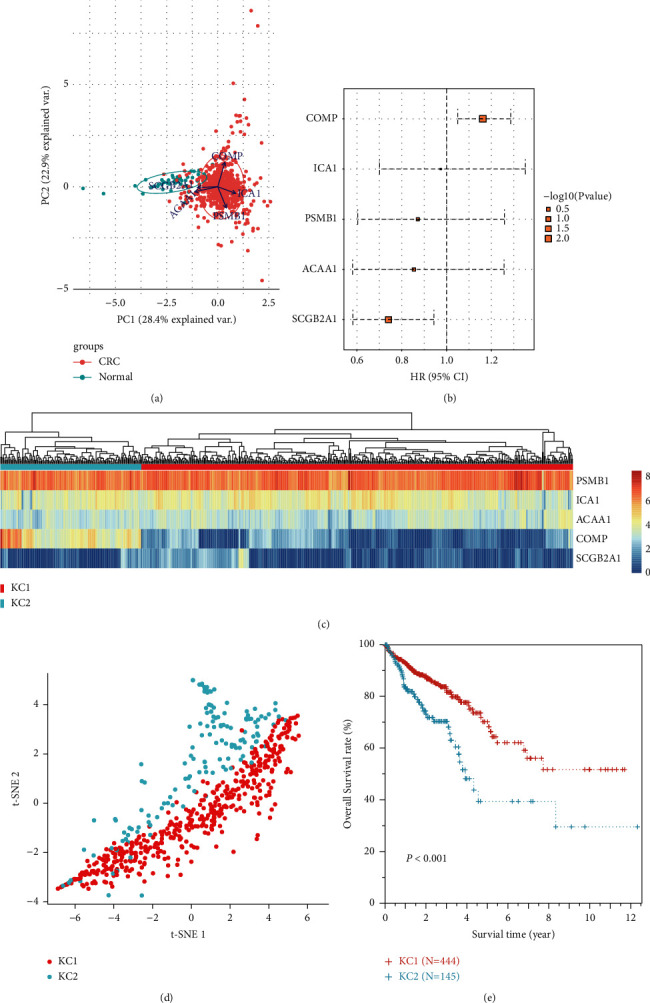
Survival analysis for the five genes in TCGA dataset. (a) Principal component analysis showing the normal and cancer samples. Arrows originating from the center point represent axes of the five genes. (b) Forest plot showing the hazard ratio (HR) of the five genes. The upper and lower error bars indicate the 95% confidence intervals. Log rank *P* values are represented by the rectangle size. (c) *K*-means clustering for CRC samples. The heatmap shows the gene expressions and the upper side bar indicates subgroups (KC1 and KC2). (d) *t*-SNE analysis showing the two subgroups of CRC samples. The points represent the samples. (e) Survival curves of KC1 and KC2 subgroups.

The association of six genes with CRC prognosis was further investigated using the TCGA dataset. *CDH19* was excluded in following analysis due to its low expression (less than 1 in more than 90% of CRC samples). Based on the expression profiles of 5 genes, PCA showed that normal and cancer samples were clearly separated, with *COMP*, *ICA1,* and *PSMB1* contributing to PC1 and *ACAA1* and with *SCGB2A1* contributing to PC2 (Figure 5(a)). Survival analysis indicated that *COMP* and *SCGB2A1* were associated with CRC prognosis (log rank *P* value <0.05), which served as poor and favorable prognostic factors, respectively (Figure 5(b)). Interestingly, CRC samples could be clustered into two subgroups, KC1 (*n* = 444) and KC2 (*n* = 145), using the *K*-means method (Figure 5(c)). The *t*-SNE analysis also revealed a large difference between KC1 and KC2 groups (Figure 5(d)). Moreover, patients of KC2 group showed worse prognosis than patients of KC1 group (Figure 5(c)).

### 3.5. GSEA of the Two Subgroups

**Figure 6:**
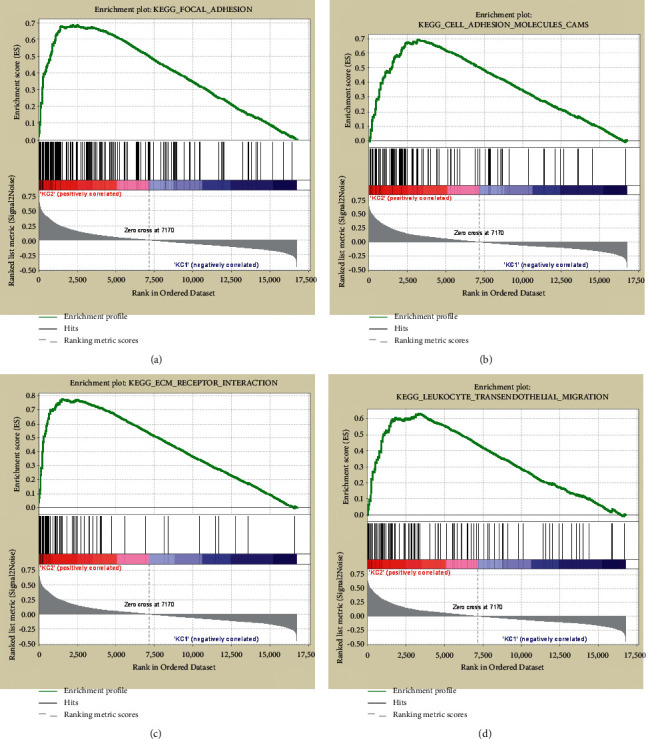
Gene set expression enrichment in KC1 and KC2 subgroups. GSEA plots showing the four significantly enriched pathways that related to cell adhesion, including FOCAL ADHESION (a), CELL ADHESION MOLECULES CAMS (b), ECM RECEPTOR INTERACTION (c), and LEUKOCYTE TRANSENDOTHELIAL MIGRATION (d).

To explain the possible biological mechanisms of the six genes, we performed gene set expression analysis to investigate the altered pathways between KC1 and KC2 subgroups. In total, we obtained 48 significantly enriched pathways (FDR <0.05), all of which showed higher scores on KC2 than KC1 (Supplementary Table 4). Cell adhesion was the predominantly enriched pathways, including FOCAL ADHESION (Figure 6(a)), CELL ADHESION MOLECULES CAMS (Figure 6(b)), ECM RECEPTOR INTERACTION (Figure 6(c)), and LEUKOCYTE TRANSENDOTHELIAL MIGRATION (Figure 6(d)), suggesting an abnormal activation of this biological process on KC2. Previous studies have revealed the tight correlation of cell adhesion with poor CRC prognosis [33, 34], which coincided with the observations of this study.

### 3.6. Developing the Classification Model of CRC

**Figure 7:**
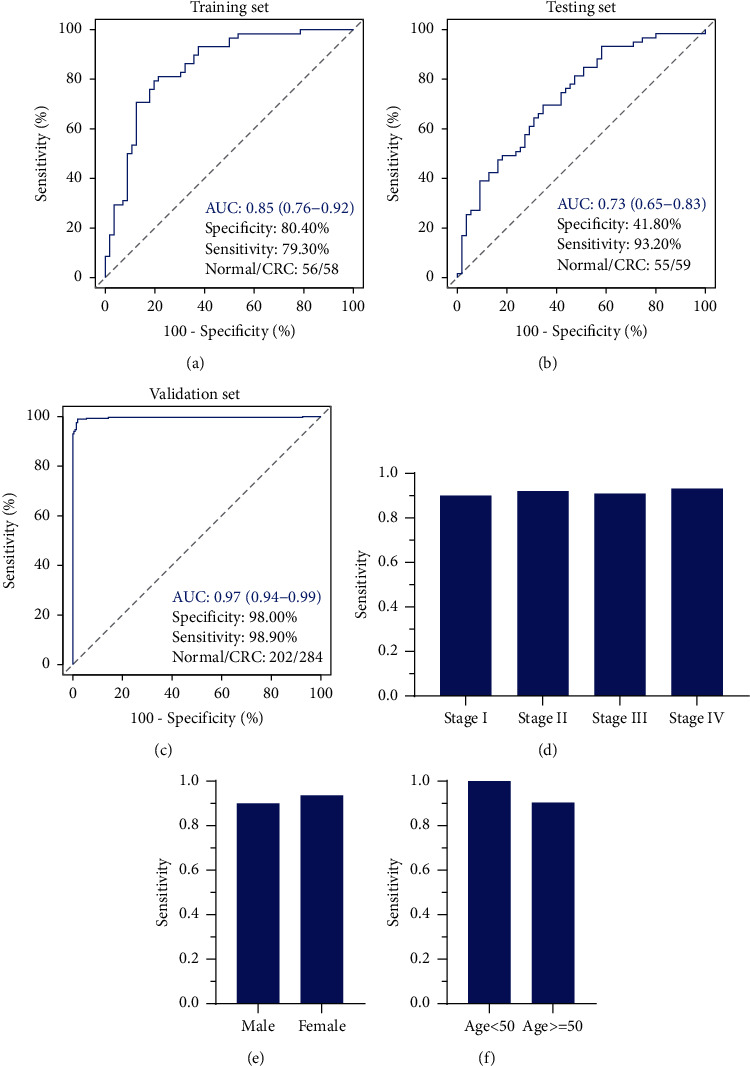
The performance of the model on training set (a), testing set (b), and validation set (c). (d)–(f): sensitivities of the model in detecting different clinical features CRCs. The sensitivity and specificity were estimated when the AUC values of the model achieved maximum.

Based on the six-gene expression profiles, we developed a classification model for CRCs using logistic regression (Table 3). The model was able to detect CRCs with a sensitivity and specificity of 79.30% and 80.40% in the training set (Figure 7(a)). The sensitivity also achieved 93.20% on the testing set, though the specificity was only 41.80% (Figure 7(b)). However, the areas under the ROC curves (AUCs) were 0.85 (0.76–0.92) and 0.73 (0.65–0.83) in the training and test sets, respectively. The sensitivity and specificity of the model even improved to 98.90% and 98.00%, with an AUC of 0.97 (0.94–0.99) in the validation set of tissue samples (Figure 7(c)). For early-stage (I + II) and advanced-stage (III + IV) CRCs, the sensitivities were 91.00% and 92.00%, respectively (Figure 7(d)). Besides, the average sensitivities were also over 90.00% for different gender and age CRCs (Figures 7(e) and 7(f)). These results demonstrated the good performance of the model in discriminating CRCs and normal samples.

### 3.7. Performance of the Six-Gene Classification Model in Adenoma Detection

**Figure 8:**
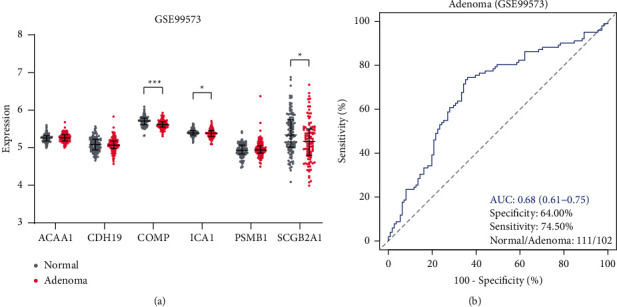
Performance of the six-gene classification model for adenomas detection. (a) Expression profiles of the six genes between normal and adenoma samples. (b) ROC curve of the six-gene classification model for the detection of adenomas.

The performance of the model for adenoma detection was further evaluated using the GSE99573 dataset. We found that three of the six genes, COMP, ICA1, and SCGB2A1, were significantly downregulated in adenoma samples (Figure 8(a)). ROC analysis showed that the sensitivity for adenomas detection was 74.50% which was a little lower than that of CRCs (Figure 8(b)). The specificity and AUC were also much lower, suggesting its inferior performance for the detection of adenomas.

### 3.8. Performance of the Six Genes for Pan-Cancer Classification

**Figure 9:**
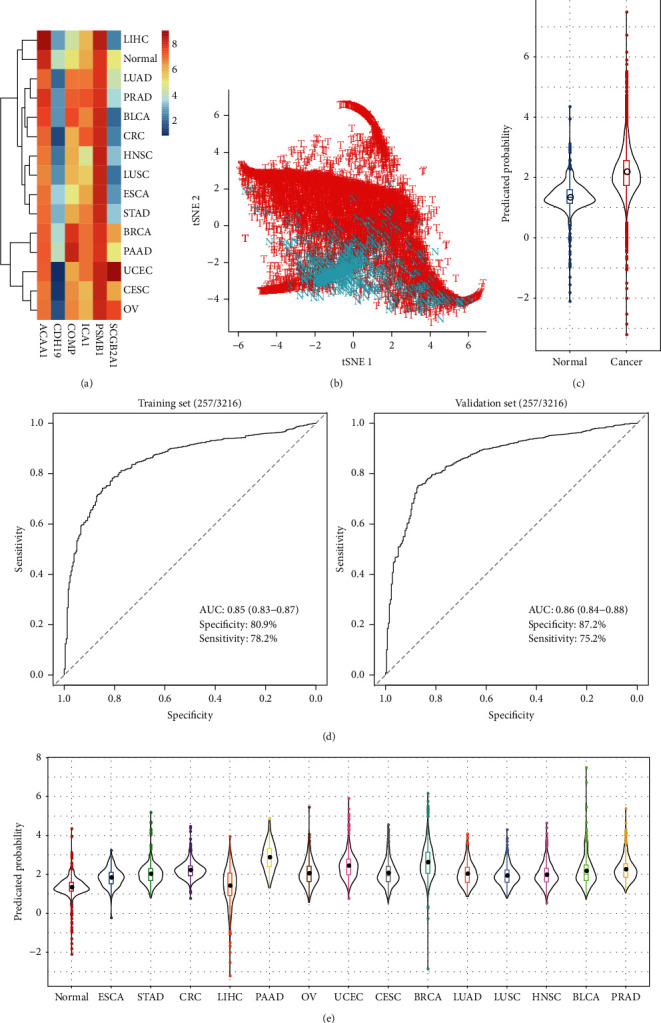
Performance of the six genes for pan-cancer classification. (a) The average expression levels of the six genes in 14 cancer types and normal samples. (b) *t*-SNE plot showing the normal and cancer samples. (c) Predicated probability of normal and cancer samples. (d) ROC curves for training and validation sets. (e) Predicated probability of normal and cancer samples across the 14 cancer types.

The performance of the six genes for other cancer classifications was further evaluated. The expression profiles of 14 most common cancers, including ESCA, STAD, CRC, LIHC, PAAD, OV, UCEC, CESC, BRCA, LUAD, LUSC, HNSC, BLCA, and PRAD, were downloaded, and the average expression levels of the six genes on each cancer type were subsequently calculated. *CDH19* showed the lowest expression in all 14 cancer types, while *PSMB1* presented the highest expression levels (Figure 9(a)). *t*-SNE analysis indicated significant differences between normal and cancer samples (Figure 9(b)), implying the potential of the six genes for pan-cancer classification. A logistic regression model was then developed based on their expression profiles, with higher predicted probabilities for pan-cancer samples than normal samples (Figure 9(c)). AUC values for training and testing sets were 0.85 (95% CI: 0.83–0.87) and 0.86 (95% CI: 0.84–0.88), respectively (Figure 9(d)). The predicated probabilities also showed significant variations among different cancer types with the highest values for PAAD and the lowest values for LIHC (Figure 9(e)). The optimal sensitivity and specificity were determined using the maximized Youden index (Table 4). The model showed the highest AUC for PAAD with sensitivity and specificity of 0.93 and 0.90. For CRC, the model showed the inferior largest AUC with sensitivity and specificity of 0.88 and 0.87. In contrast, the smallest AUC was observed for LIHC with sensitivity and specificity of 0.42 and 0.84.

## 4. Discussion

In this study, we integrated two independent datasets to identify 1841 DEGs between CRC and matched normal samples, of which 1065 were upregulated and 776 were downregulated in tumor samples. Functional annotations revealed that the DEGs were mainly involved in the biological processes of extracellular structure and biosynthetic process. Six genes selected by LASSO regression were then used to develop a CRC classification model, which we called the CRC classifier. The classifier showed good performance on training and testing sets of stool samples, as well as the CRC tissue samples. These results indicated the potential utility of the classifier for CRC detection.

The current study integrated two independent datasets to identify 1841 common DEGs significantly enriched in the extracellular matrix (ECM), which represented the possibly disrupted biological pathway in the development of colorectal cancer. ECM is the principal structure of tumor microenvironment, and the components can regulate cell and tissue morphology and structure by interacting with cell surface receptors, transcription factors, and cytokines. Therefore, ECM is tightly associated with the development and progression of cancer cells [35]. Furthermore, ECM was identified in several studies to be significantly enriched by the DEGs between normal samples and CRCs [36, 37]. Besides, GSEA results also indicated higher enrichment scores of ECM receptor interaction pathway in KC2 group samples. Given the worse prognosis of KC2 than KC1, findings of this study demonstrated the potential key role of ECM in colorectal cancer, which would provide a new path to study the underlying mechanism of this disease further.

In TCGA dataset, survival analysis indicated that *COMP* and *SCGB2A1* were associated with CRC prognosis (*P* < 0.05). Interestingly, TCGA CRCs clustered into two subgroups (KC1 and KC2) based on expression profiles of the five genes, and the KC2 subgroup showed worse prognosis than KC1 subgroup. We observed higher expression of the poor prognostic gene *COMP* on KC2, while it was the opposite for the favorable prognostic gene *SCGB2A1*, which might interpret the different prognosis between the two subgroups. *COMP* was found overexpressed on early-set CRC patients and associated with poor CRC prognosis, which was consistent with our findings [38]. *SCGB2A1* was also reported as a prognostic factor for CRC [39].

Further investigations revealed that the genes *CDH19* and *SCGB2A1* were reported to be downregulated in CRC tissues in previous studies [39–41], which is consistent with our results. The overexpression of *ACAA1* was reported in CRC tissues, while it was the opposite in this study, which we infer might be affected by the complex background noise of the fecal samples [42]. In addition, *ICA1* and *PSMB1* are two genes described for the first time in this study capable of the CRC classification. The average sensitivity of the classifier for CRC stool samples is around 85% which is better than that of gFOBT/FIT, although the average specificity is inferior to the gFOBT/FIT test.

The ability to detect early-stage CRCs is an important property of a diagnostic classifier. The current classifier obtained a sensitivity of 91.00% for stage I-II CRCs, which was close to the stage III-IV CRCs, suggesting its good utility in detecting early-stage CRCs. Further analysis revealed that the six genes were already upregulated in early-stage CRCs, which might explain the well-performed classifier for the early detection of CRC. Furthermore, the sensitivities of this classifier in all CRC samples stratified by patient age and gender did not show significant variations, indicating its capability was not affected by the clinical features. Altogether, the classifier showed a robust performance for CRC early detection.

Adenomas are recognized as the precancerous lesions of CRC. Patients with adenomas have a higher risk than the average population to develop cancer [43], and thus early detection of adenomas can effectively reduce the incidence of CRC. Here, the sensitivity of this classifier for adenoma detection was 74.50%, which was superior to gFOBT/FIT, with an AUC of 0.68 (95% CI: 0.61–0.75) [44, 45]. Barnell et al. developed stool-derived eukaryotic RNA (seRNA) sequences as biomarkers that showed an AUC of 0.70 whereby high-risk adenomas (HRAs) were considered positive and other findings (medium-risk adenomas, low-risk adenomas, benign polyps, no findings on a colonoscopy) were considered negative [26]. These results demonstrate the good ability of this classifier in detecting adenomas and could serve as a potential strategy to reduce CRC incidence. However, considering the high complexity and vast differences of histological subtypes in adenomas (for example, villous adenomas showed higher risk than tubular adenomas [46]), further investigations in the future are needed to evaluate the classifier comprehensively. Compared with the above biomarkers, our six-gene signature outperformed the abovementioned biomarkers, with an AUC of 0.85, 0.73, and 0.97 in the training, testing, and validation sets where the training and testing sets belong to GSE99573 dataset uploaded by Barnell et al. [26].

The tumor and normal samples of 14 cancer types showed significant differences based on the expression profiles of 6 genes revealed by t-SNE analysis. AUC values for both training and testing sets exceeded 0.85, suggesting the potential ability of the 6-gene signature for pan-cancer detection. However, for liver cancer samples, the AUC was only 0.55, which was the lowest among the 14 cancer types, implying the variations of the six genes across different cancers.

In summary, the current study provided a six gene-based classifier that showed good performance for the classification of CRCs by integrating multiple datasets. However, several issues remain to be addressed. Firstly, the classifier needs to be validated in additional external studies of fecal samples to evaluate the real clinical performance. Secondly, the specificity for CRC detection was less than 80% which should be improved for further work. Besides, it is still essential to investigate the practice of the classifier for the detection of precancerous lesions such as adenomas and polyps. Nevertheless, our results provide six novel mRNAs as the candidates for CRC early detection, which expands the diversity of biomarker resources, especially the inadequate mRNA repertoire. Moreover, the developed classifier showed an ability to discriminate cancer and normal samples across multiple cancer types, suggesting its potential as pan-cancer detection markers.

## Figures and Tables

**Table 1:** Datasets used in this study.

Dataset	Normal		Adenoma	Tumor	Platform	Sample type
	Mucosa	Adjacent				
GSE106582	68			68	GPL10558	Tissue
GSE117606	57		57	57	GPL25373	Tissue
GSE44076	50	98		98	GPL13667	Tissue
GSE41258		54		189	GPL96	Tissue
GSE99573		111	102	117	GPL17586	Stool

**Table 2:** Clinical features of the subjects in GSE99573 dataset.

Features	Normal (*n*, %)	Adenoma (*n*, %)	CRC (*n*, %)
Gender (*n*, %)			
Male	66 (33.85%)	59 (30.26%)	70 (35.90%)
Female	45 (33.33%)	43 (31.85%)	47 (34.81%)

Age (*n*, %)			
<50	24 (35.29%)	31 (45.59%)	13 (19.12%)
≥50	87 (33.21%)	71 (27.10%)	104 (39.69%)

AJCC stage (*n*, %)			
Stage I			30 (25.64%)
Stage II			25 (21.37%)
Stage III			33 (28.21%)
Stage IV			29 (24.79%)
Total (*n*, %)	111 (100%)	102 (100%)	117 (100.00%)

**Table 3:** Coefficients of the six genes developed by logistic regression.

	Intercept	*ACAA1*	*CDH19*	*COMP*	*ICA1*	*PSMB1*	*SCGB2A1*
Coefficient	41.85	6.82	−4.04	−4.94	−7.85	3.42	−0.72

**Table 4:** The optimal sensitivities and specificities for 14 cancer types.

Tumor code	Sensitivity (%, 95% CI)	Specificity (%, 95% CI)	AUC (95% CI)
ESCA	65.22 (58.34–72.10)	81.52 (78.16–84.87)	0.79 (0.76∼0.83)
STAD	82.65 (79.01–86.29)	74.12 (70.34–77.91)	0.85 (0.82∼0.87)
CRC	88.16 (85.59–90.73)	86.96 (84.05–89.88)	0.93 (0.91∼0.94)
LIHC	41.51 (36.50–46.52)	84.44 (81.30–87.57)	0.55 (0.51∼0.59)
PAAD	92.7 (88.87–96.52)	90.47 (87.93–93.01)	0.97 (0.96∼0.98)
OV	76.97 (72.24–81.71)	75.29 (71.56–79.02)	0.81 (0.78∼0.84)
UCEC	85.34 (82.33–88.34)	86.96 (84.05–89.88)	0.92 (0.90∼0.94)
CESC	68.75 (63.54–73.96)	84.63 (81.51–87.75)	0.82 (0.78∼0.84)
BRCA	88.13 (86.21–90.04)	86.96 (84.05–89.88)	0.93 (0.92∼0.94)
LUAD	67.57 (63.53–71.62)	84.63 (81.51–87.75)	0.82 (0.79∼0.84)
LUSC	64.47 (60.28–68.66)	84.44 (81.30–87.57)	0.80 (0.77∼0.82)
HNSC	75.38 (71.68–79.09)	74.12 (70.34–77.91)	0.80 (0.77∼0.82)
BLCA	74.75 (70.54–78.97)	80.93 (77.54–84.33)	0.84 (0.81∼0.86)
PRAD	83.1 (79.80–86.39)	84.24 (81.09–87.39)	0.91 (0.88∼0.92)

## Data Availability

The datasets analyzed in this study could be found in [GSE106582] at https://www.ncbi.nlm.nih.gov/geo/query/acc.cgi?acc=GSE106582 and in [GSE117606] at https://www.ncbi.nlm.nih.gov/geo/query/acc.cgi?acc=GSE117606.

## Supplementary Materials

Supplementary Materials Supplementary Table 1. The samples of 14 cancer types. Supplementary Table 2. The top 10% overlapped DEGs (*n* = 184) between GSE106582 and GSE117606 datasets. Supplementary Table 3. Top 10% enriched GO terms (*n* = 80) of the overlapped DEGs. Supplementary Table 4. Enriched KEGG pathways of the overlapped DEGs. Supplementary Table 5. Top 20 enrichments of GSEA in KC2 subgroup.
