# Supplementary material for: Identification of Six Genes as Diagnostic Markers for Colorectal Cancer Detection by Integrating Multiple Expression Profiles
Source: J Oncol. 2022 Jul 22;2022:3850674. doi: 10.1155/2022/3850674 (PMC9337943; doi:10.1155/2022/3850674)
Supplement: Supplementary Materials — Supplementary Table 1. The samples of 14 cancer types. Supplementary Table 2. The top 10% overlapped DEGs (n = 184) between GSE106582 and GSE117606 datasets. Supplementary Table 3. Top 10% enriched GO terms (n = 80) of the overlapped DEGs. Supplementary Table 4. Enriched KEGG pathways of the overlapped DEGs. Supplementary Table 5. Top 20 enrichments of GSEA in KC2 subgroup. [file 3850674.f1.docx]

## ****Supplementary tables****

**Supplementary table 1.** The sample sizes of 14 cancer types.

| Tumor code | Cancer (n) | Normal (n) |
| --- | --- | --- |
| ESCA | 184 | 11 |
| STAD | 415 | 35 |
| COAD | 449 | 41 |
| READ | 159 | 10 |
| LIHC | 371 | 50 |
| PAAD | 178 | 4 |
| OV | 304 | 0 |
| UCEC | 532 | 22 |
| CESC | 304 | 3 |
| BRCA | 1095 | 113 |
| LUAD | 515 | 59 |
| LUSC | 501 | 51 |
| HNSC | 520 | 44 |
| BLCA | 408 | 19 |
| PRAD | 497 | 52 |
| Total | 6432 | 514 |

**Supplementary table 2.** The top 10% overlapped DEGs (n=184) between GSE106582 and GSE117606 datasets.

| gName | GSE106582 | | | GSE117606 | | | Regulated |
| --- | --- | --- | --- | --- | --- | --- | --- |
|  | log(Fold change) | P.value | FDR | log(Fold change) | P.value | FDR |  |
| AARS1 | 1.0437 | 8.47E-16 | 3.86E-11 | 1.064 | 1.12E-08 | 0.0002 | Up |
| AASDHPPT | 1.0349 | 1.03E-11 | 4.53E-07 | 1.161 | 2.20E-07 | 0.0038 | Up |
| ABCC1 | 1.0332 | 7.12E-13 | 3.17E-08 | 1.143 | 4.44E-11 | 0.0000 | Up |
| ABCE1 | 1.0891 | 8.91E-22 | 4.18E-17 | 1.145 | 4.80E-10 | 0.0000 | Up |
| ABHD2 | 1.0405 | 9.02E-11 | 3.90E-06 | 1.135 | 6.41E-12 | 0.0000 | Up |
| ACBD6 | 1.0489 | 1.47E-16 | 6.75E-12 | 1.117 | 5.47E-07 | 0.0095 | Up |
| ACD | 1.0598 | 3.74E-20 | 1.74E-15 | 1.160 | 6.34E-07 | 0.0109 | Up |
| ACLY | 1.0512 | 1.60E-12 | 7.08E-08 | 1.115 | 1.30E-14 | 0.0000 | Up |
| ACP1 | 1.0432 | 1.26E-13 | 5.65E-09 | 1.099 | 2.35E-12 | 0.0000 | Up |
| ACSL6 | 1.0247 | 7.06E-07 | 0.028045 | 1.455 | 2.44E-06 | 0.0412 | Up |
| ADAT2 | 1.0318 | 3.42E-10 | 1.47E-05 | 1.178 | 1.07E-07 | 0.0019 | Up |
| ADGRF5 | 1.0519 | 2.72E-07 | 0.010924 | 1.174 | 2.27E-06 | 0.0384 | Up |
| ADGRG1 | 1.0903 | 4.72E-14 | 2.12E-09 | 1.163 | 7.93E-16 | 0.0000 | Up |
| ADK | 1.0735 | 7.04E-15 | 3.19E-10 | 1.137 | 2.34E-08 | 0.0004 | Up |
| ADNP | 1.0557 | 1.54E-19 | 7.15E-15 | 1.107 | 2.26E-08 | 0.0004 | Up |
| ADRM1 | 1.0961 | 3.90E-27 | 1.84E-22 | 1.088 | 1.29E-09 | 0.0000 | Up |
| AGAP3 | 1.0667 | 1.74E-19 | 8.11E-15 | 1.104 | 3.83E-09 | 0.0001 | Up |
| AGO2 | 1.0486 | 7.92E-08 | 0.003227 | 1.117 | 4.39E-10 | 0.0000 | Up |
| AGRN | 1.0586 | 6.14E-11 | 2.67E-06 | 1.102 | 2.07E-09 | 0.0000 | Up |
| AGT | 1.1245 | 1.58E-13 | 7.07E-09 | 1.219 | 4.41E-07 | 0.0076 | Up |
| AGTPBP1 | 1.0407 | 8.43E-10 | 3.59E-05 | 1.127 | 1.15E-06 | 0.0196 | Up |
| AGTRAP | 1.0650 | 2.31E-15 | 1.05E-10 | 1.114 | 6.67E-08 | 0.0012 | Up |
| AHCY | 1.1007 | 1.72E-24 | 8.11E-20 | 1.220 | 6.30E-12 | 0.0000 | Up |
| AJUBA | 1.0691 | 7.02E-18 | 3.24E-13 | 1.165 | 2.03E-06 | 0.0344 | Up |
| ALG5 | 1.0393 | 2.87E-09 | 0.000121 | 1.118 | 2.89E-06 | 0.0487 | Up |
| ALG8 | 1.0440 | 9.55E-12 | 4.19E-07 | 1.244 | 1.13E-08 | 0.0002 | Up |
| ALYREF | 1.0557 | 1.09E-10 | 4.73E-06 | 1.099 | 2.57E-09 | 0.0000 | Up |
| ANGPT2 | 1.1045 | 3.29E-20 | 1.53E-15 | 1.168 | 3.29E-07 | 0.0057 | Up |
| ANKLE2 | 1.0575 | 2.41E-14 | 1.09E-09 | 1.084 | 3.63E-09 | 0.0001 | Up |
| ANKRD22 | 1.0695 | 6.25E-07 | 0.024856 | 1.187 | 9.40E-09 | 0.0002 | Up |
| ANLN | 1.1650 | 3.41E-25 | 1.61E-20 | 1.447 | 1.59E-15 | 0.0000 | Up |
| ANTXR1 | 1.0834 | 1.05E-13 | 4.73E-09 | 1.134 | 2.95E-06 | 0.0498 | Up |
| ANXA3 | 1.1227 | 3.71E-16 | 1.70E-11 | 1.331 | 3.77E-14 | 0.0000 | Up |
| AP1S1 | 1.0660 | 5.91E-11 | 2.56E-06 | 1.100 | 2.93E-09 | 0.0001 | Up |
| AP2S1 | 1.0271 | 6.26E-07 | 0.024916 | 1.060 | 3.91E-11 | 0.0000 | Up |
| APEX1 | 1.0313 | 2.55E-09 | 0.000108 | 1.056 | 2.14E-06 | 0.0363 | Up |
| APMAP | 1.0683 | 6.39E-14 | 2.87E-09 | 1.112 | 1.38E-09 | 0.0000 | Up |
| ARHGAP39 | 1.0515 | 6.72E-10 | 2.87E-05 | 1.214 | 5.65E-07 | 0.0098 | Up |
| ARID3A | 1.1302 | 3.22E-14 | 1.45E-09 | 1.144 | 3.99E-07 | 0.0069 | Up |
| ARL5B | 1.0535 | 6.53E-08 | 0.002666 | 1.128 | 9.36E-07 | 0.0160 | Up |
| ARMC10 | 1.0804 | 3.30E-23 | 1.55E-18 | 1.218 | 1.73E-08 | 0.0003 | Up |
| ASAP1 | 1.0473 | 3.43E-10 | 1.47E-05 | 1.153 | 6.15E-10 | 0.0000 | Up |
| ASCC3 | 1.0420 | 2.77E-11 | 1.21E-06 | 1.167 | 1.14E-08 | 0.0002 | Up |
| ASCL2 | 1.2523 | 1.87E-20 | 8.74E-16 | 1.519 | 4.10E-15 | 0.0000 | Up |
| ASPH | 1.0372 | 1.44E-08 | 0.000596 | 1.167 | 1.98E-13 | 0.0000 | Up |
| ASPM | 1.1199 | 5.16E-15 | 2.34E-10 | 1.309 | 3.00E-11 | 0.0000 | Up |
| ASPSCR1 | 1.0539 | 4.58E-07 | 0.018278 | 1.091 | 1.62E-07 | 0.0028 | Up |
| ASXL1 | 1.0547 | 7.39E-15 | 3.35E-10 | 1.098 | 2.64E-06 | 0.0446 | Up |
| ATAD2 | 1.1155 | 7.07E-20 | 3.29E-15 | 1.298 | 1.01E-13 | 0.0000 | Up |
| ATIC | 1.0616 | 5.08E-21 | 2.38E-16 | 1.108 | 1.98E-16 | 0.0000 | Up |
| ATP2C1 | 1.0229 | 2.21E-07 | 0.0089 | 1.146 | 7.21E-07 | 0.0124 | Up |
| ATP6V1C1 | 1.0237 | 8.18E-10 | 3.49E-05 | 1.093 | 2.79E-06 | 0.0472 | Up |
| ATP6V1F | 1.0754 | 1.64E-24 | 7.75E-20 | 1.119 | 1.63E-11 | 0.0000 | Up |
| ATP6V1G1 | 1.0236 | 9.61E-08 | 0.003908 | 1.075 | 5.50E-07 | 0.0095 | Up |
| AURKA | 1.1567 | 1.33E-21 | 6.25E-17 | 1.275 | 6.32E-09 | 0.0001 | Up |
| AXIN2 | 1.1398 | 1.02E-14 | 4.61E-10 | 1.348 | 2.23E-13 | 0.0000 | Up |
| AZGP1 | 1.1267 | 3.87E-15 | 1.76E-10 | 1.810 | 3.97E-14 | 0.0000 | Up |
| AZIN1 | 1.0319 | 3.18E-07 | 0.012766 | 1.220 | 2.38E-10 | 0.0000 | Up |
| B3GALT6 | 1.0298 | 8.17E-08 | 0.003328 | 1.134 | 4.23E-09 | 0.0001 | Up |
| BACE2 | 1.0852 | 1.97E-12 | 8.73E-08 | 1.237 | 2.85E-11 | 0.0000 | Up |
| BANF1 | 1.0416 | 3.97E-13 | 1.77E-08 | 1.057 | 5.68E-08 | 0.0010 | Up |
| BCAP31 | 1.0471 | 1.81E-15 | 8.25E-11 | 1.097 | 8.09E-09 | 0.0001 | Up |
| BCL2L1 | 1.0499 | 4.38E-12 | 1.93E-07 | 1.143 | 2.02E-14 | 0.0000 | Up |
| BCL2L12 | 1.1092 | 7.18E-24 | 3.38E-19 | 1.135 | 3.63E-09 | 0.0001 | Up |
| BGN | 1.2179 | 4.14E-23 | 1.95E-18 | 1.368 | 8.83E-14 | 0.0000 | Up |
| BHLHE40 | 1.0756 | 1.91E-10 | 8.21E-06 | 1.177 | 4.06E-15 | 0.0000 | Up |
| BID | 1.0370 | 9.30E-09 | 0.000388 | 1.127 | 1.55E-09 | 0.0000 | Up |
| BIRC5 | 1.1278 | 1.92E-16 | 8.79E-12 | 1.218 | 1.39E-08 | 0.0002 | Up |
| BLM | 1.0536 | 7.43E-13 | 3.30E-08 | 1.250 | 7.16E-07 | 0.0123 | Up |
| BOP1 | 1.0274 | 1.59E-09 | 6.73E-05 | 1.162 | 1.21E-09 | 0.0000 | Up |
| BORA | 1.0998 | 2.11E-16 | 9.65E-12 | 1.321 | 4.73E-07 | 0.0082 | Up |
| BRCA1 | 1.0575 | 1.44E-12 | 6.37E-08 | 1.163 | 2.65E-07 | 0.0046 | Up |
| BRCA2 | 1.0253 | 1.19E-09 | 5.04E-05 | 1.238 | 4.85E-07 | 0.0084 | Up |
| BRIX1 | 1.1008 | 2.63E-24 | 1.24E-19 | 1.179 | 1.16E-10 | 0.0000 | Up |
| BUB1 | 1.1386 | 2.03E-20 | 9.46E-16 | 1.229 | 8.34E-10 | 0.0000 | Up |
| BUB3 | 1.0326 | 4.59E-13 | 2.05E-08 | 1.098 | 3.36E-12 | 0.0000 | Up |
| BUD23 | 1.0613 | 1.89E-17 | 8.72E-13 | 1.153 | 1.94E-09 | 0.0000 | Up |
| BYSL | 1.1054 | 3.23E-27 | 1.53E-22 | 1.271 | 3.38E-12 | 0.0000 | Up |
| BZW2 | 1.0589 | 4.82E-16 | 2.20E-11 | 1.116 | 1.04E-09 | 0.0000 | Up |
| C19orf53 | 1.0647 | 1.07E-22 | 5.04E-18 | 1.070 | 1.81E-12 | 0.0000 | Up |
| C1GALT1 | 1.0607 | 2.63E-11 | 1.15E-06 | 1.092 | 2.95E-07 | 0.0051 | Up |
| C2 | 1.1322 | 9.27E-23 | 4.35E-18 | 1.136 | 1.02E-07 | 0.0018 | Up |
| C2CD4A | 1.0493 | 7.62E-09 | 0.000318 | 1.911 | 3.32E-08 | 0.0006 | Up |
| C4orf48 | 1.0787 | 1.60E-13 | 7.15E-09 | 1.139 | 4.11E-07 | 0.0071 | Up |
| C8orf33 | 1.0571 | 2.94E-08 | 0.001209 | 1.193 | 9.77E-09 | 0.0002 | Up |
| C9orf16 | 1.0627 | 1.12E-15 | 5.10E-11 | 1.144 | 1.86E-08 | 0.0003 | Up |
| CA9 | 1.1969 | 1.81E-13 | 8.12E-09 | 1.207 | 1.40E-06 | 0.0239 | Up |
| CAD | 1.0765 | 2.87E-21 | 1.34E-16 | 1.148 | 3.11E-11 | 0.0000 | Up |
| CALU | 1.0962 | 3.62E-15 | 1.65E-10 | 1.097 | 3.53E-10 | 0.0000 | Up |
| CAPG | 1.0491 | 1.50E-08 | 0.000622 | 1.119 | 3.19E-10 | 0.0000 | Up |
| CAPRIN1 | 1.0570 | 7.16E-15 | 3.24E-10 | 1.080 | 8.32E-08 | 0.0015 | Up |
| CARHSP1 | 1.0826 | 1.83E-17 | 8.45E-13 | 1.117 | 4.09E-09 | 0.0001 | Up |
| CASK | 1.0402 | 1.22E-06 | 0.048084 | 1.106 | 6.67E-09 | 0.0001 | Up |
| CASP2 | 1.0472 | 4.08E-12 | 1.80E-07 | 1.125 | 3.19E-14 | 0.0000 | Up |
| CBFB | 1.0942 | 4.63E-25 | 2.18E-20 | 1.222 | 5.39E-16 | 0.0000 | Up |
| CBX2 | 1.1540 | 6.60E-19 | 3.06E-14 | 1.182 | 5.95E-07 | 0.0103 | Up |
| CBX3 | 1.0753 | 1.64E-23 | 7.70E-19 | 1.166 | 3.00E-15 | 0.0000 | Up |
| CBX4 | 1.0920 | 2.22E-18 | 1.03E-13 | 1.228 | 8.16E-13 | 0.0000 | Up |
| CCDC43 | 1.0271 | 3.33E-08 | 0.001371 | 1.108 | 2.37E-06 | 0.0400 | Up |
| CCDC59 | 1.0419 | 4.11E-15 | 1.87E-10 | 1.101 | 1.27E-07 | 0.0022 | Up |
| CCDC86 | 1.1299 | 1.07E-30 | 5.04E-26 | 1.173 | 1.82E-14 | 0.0000 | Up |
| CCN4 | 1.1101 | 3.79E-19 | 1.76E-14 | 1.541 | 3.38E-11 | 0.0000 | Up |
| CCNA2 | 1.1184 | 5.63E-16 | 2.57E-11 | 1.128 | 3.47E-08 | 0.0006 | Up |
| CCNB1 | 1.1187 | 1.92E-15 | 8.75E-11 | 1.316 | 4.98E-11 | 0.0000 | Up |
| CCNB1IP1 | 1.0656 | 1.42E-12 | 6.30E-08 | 1.104 | 5.16E-11 | 0.0000 | Up |
| CCNB2 | 1.0995 | 2.56E-11 | 1.12E-06 | 1.312 | 3.41E-07 | 0.0059 | Up |
| CCND1 | 1.0913 | 4.71E-19 | 2.19E-14 | 1.211 | 5.96E-14 | 0.0000 | Up |
| CCNF | 1.1407 | 7.71E-23 | 3.62E-18 | 1.147 | 2.85E-06 | 0.0481 | Up |
| CCT2 | 1.0777 | 2.92E-23 | 1.37E-18 | 1.148 | 1.33E-16 | 0.0000 | Up |
| CCT3 | 1.0766 | 6.88E-25 | 3.24E-20 | 1.107 | 1.48E-15 | 0.0000 | Up |
| CCT4 | 1.0628 | 1.25E-19 | 5.82E-15 | 1.128 | 9.12E-12 | 0.0000 | Up |
| CCT5 | 1.0470 | 1.59E-12 | 7.06E-08 | 1.111 | 5.75E-09 | 0.0001 | Up |
| CCT6A | 1.0861 | 7.77E-24 | 3.66E-19 | 1.222 | 1.25E-13 | 0.0000 | Up |
| CCT7 | 1.0613 | 3.03E-23 | 1.43E-18 | 1.086 | 3.56E-08 | 0.0006 | Up |
| CCT8 | 1.0497 | 5.33E-19 | 2.47E-14 | 1.178 | 6.18E-13 | 0.0000 | Up |
| CD44 | 1.0485 | 1.10E-14 | 4.99E-10 | 1.161 | 2.67E-16 | 0.0000 | Up |
| CD46 | 1.0682 | 1.23E-15 | 5.61E-11 | 1.150 | 4.88E-16 | 0.0000 | Up |
| CD55 | 1.1036 | 3.51E-09 | 0.000148 | 1.147 | 4.89E-07 | 0.0085 | Up |
| CDC123 | 1.0492 | 3.13E-15 | 1.42E-10 | 1.086 | 1.31E-08 | 0.0002 | Up |
| CDC16 | 1.0419 | 1.37E-13 | 6.12E-09 | 1.070 | 9.86E-07 | 0.0169 | Up |
| CDC25B | 1.1880 | 1.66E-29 | 7.85E-25 | 1.170 | 5.64E-15 | 0.0000 | Up |
| CDC5L | 1.0271 | 9.56E-09 | 0.000398 | 1.063 | 7.53E-08 | 0.0013 | Up |
| CDC6 | 1.0189 | 5.54E-07 | 0.022049 | 1.224 | 8.70E-08 | 0.0015 | Up |
| CDCA3 | 1.1162 | 3.09E-16 | 1.42E-11 | 1.141 | 1.57E-06 | 0.0268 | Up |
| CDCA5 | 1.1626 | 1.30E-20 | 6.07E-16 | 1.250 | 2.14E-10 | 0.0000 | Up |
| CDCA7 | 1.1194 | 2.77E-12 | 1.23E-07 | 1.433 | 2.21E-14 | 0.0000 | Up |
| CDH11 | 1.1380 | 1.02E-15 | 4.64E-11 | 1.286 | 5.53E-12 | 0.0000 | Up |
| CDH3 | 1.2553 | 1.25E-28 | 5.89E-24 | 1.488 | 3.09E-17 | 0.0000 | Up |
| CDK1 | 1.1345 | 2.08E-17 | 9.57E-13 | 1.276 | 1.01E-09 | 0.0000 | Up |
| CDK2 | 1.0799 | 7.77E-18 | 3.59E-13 | 1.097 | 1.95E-06 | 0.0332 | Up |
| CDK4 | 1.1013 | 7.15E-25 | 3.37E-20 | 1.163 | 7.74E-17 | 0.0000 | Up |
| CDK5 | 1.0597 | 2.54E-16 | 1.16E-11 | 1.193 | 1.86E-10 | 0.0000 | Up |
| CDK7 | 1.0617 | 2.03E-15 | 9.22E-11 | 1.217 | 2.18E-09 | 0.0000 | Up |
| CDKN3 | 1.1350 | 1.69E-18 | 7.82E-14 | 1.345 | 1.16E-12 | 0.0000 | Up |
| CEBPB | 1.0879 | 7.85E-19 | 3.64E-14 | 1.111 | 4.95E-11 | 0.0000 | Up |
| CEL | 1.2344 | 6.07E-12 | 2.67E-07 | 1.630 | 1.01E-06 | 0.0174 | Up |
| CELSR3 | 1.1609 | 4.71E-21 | 2.20E-16 | 1.264 | 1.47E-10 | 0.0000 | Up |
| CEMIP | 1.4390 | 3.52E-33 | 1.67E-28 | 1.763 | 3.08E-19 | 0.0000 | Up |
| CENPA | 1.0759 | 1.58E-17 | 7.29E-13 | 1.289 | 3.07E-07 | 0.0053 | Up |
| CENPF | 1.1095 | 3.18E-17 | 1.46E-12 | 1.283 | 2.07E-09 | 0.0000 | Up |
| CENPH | 1.0505 | 2.23E-13 | 9.95E-09 | 1.336 | 1.14E-09 | 0.0000 | Up |
| CENPJ | 1.0680 | 7.15E-12 | 3.14E-07 | 1.187 | 1.04E-07 | 0.0018 | Up |
| CENPN | 1.1318 | 5.66E-22 | 2.65E-17 | 1.228 | 4.42E-11 | 0.0000 | Up |
| CENPW | 1.1202 | 1.67E-17 | 7.68E-13 | 1.348 | 1.83E-10 | 0.0000 | Up |
| CEP55 | 1.1590 | 1.88E-20 | 8.78E-16 | 1.333 | 1.65E-16 | 0.0000 | Up |
| CEP72 | 1.0486 | 3.00E-12 | 1.32E-07 | 1.288 | 3.37E-09 | 0.0001 | Up |
| CFB | 1.1737 | 8.14E-20 | 3.79E-15 | 1.241 | 2.26E-10 | 0.0000 | Up |
| CFI | 1.1026 | 8.70E-12 | 3.82E-07 | 1.310 | 8.23E-08 | 0.0015 | Up |
| CHAF1A | 1.0434 | 3.52E-12 | 1.55E-07 | 1.129 | 1.52E-08 | 0.0003 | Up |
| CHD7 | 1.0493 | 1.01E-08 | 0.000423 | 1.095 | 6.24E-09 | 0.0001 | Up |
| CHEK1 | 1.1168 | 8.01E-20 | 3.73E-15 | 1.222 | 6.07E-11 | 0.0000 | Up |
| CHI3L1 | 1.1647 | 3.05E-18 | 1.41E-13 | 1.771 | 1.90E-16 | 0.0000 | Up |
| CHPF | 1.1031 | 5.67E-21 | 2.65E-16 | 1.124 | 1.04E-09 | 0.0000 | Up |
| CKAP2 | 1.1208 | 4.54E-23 | 2.14E-18 | 1.273 | 2.90E-12 | 0.0000 | Up |
| CKAP5 | 1.0228 | 1.23E-06 | 0.048687 | 1.079 | 4.98E-09 | 0.0001 | Up |
| CKS2 | 1.1581 | 6.50E-23 | 3.05E-18 | 1.286 | 5.42E-14 | 0.0000 | Up |
| CLDN1 | 1.4225 | 3.01E-35 | 1.42E-30 | 1.855 | 2.35E-18 | 0.0000 | Up |
| CLDN2 | 1.2351 | 9.90E-13 | 4.39E-08 | 2.326 | 4.99E-15 | 0.0000 | Up |
| CLEC5A | 1.0479 | 1.61E-11 | 7.05E-07 | 1.347 | 1.28E-08 | 0.0002 | Up |
| CLIC1 | 1.0391 | 1.29E-11 | 5.65E-07 | 1.057 | 3.88E-12 | 0.0000 | Up |
| CLIC3 | 1.0665 | 1.21E-06 | 0.047889 | 1.258 | 1.50E-07 | 0.0026 | Up |
| CLNS1A | 1.0441 | 2.10E-15 | 9.55E-11 | 1.140 | 5.48E-09 | 0.0001 | Up |
| CLTA | 1.0330 | 5.05E-10 | 2.16E-05 | 1.050 | 2.07E-07 | 0.0036 | Up |
| CMTM7 | 1.0356 | 4.75E-10 | 2.03E-05 | 1.231 | 6.50E-10 | 0.0000 | Up |
| CMTM8 | 1.0758 | 4.72E-12 | 2.08E-07 | 1.304 | 3.90E-07 | 0.0068 | Up |
| CNN2 | 1.1050 | 3.23E-23 | 1.52E-18 | 1.161 | 2.28E-14 | 0.0000 | Up |
| CNOT9 | 1.0463 | 2.62E-13 | 1.17E-08 | 1.076 | 2.25E-10 | 0.0000 | Up |
| CNPY2 | 1.0502 | 2.14E-13 | 9.58E-09 | 1.094 | 2.30E-10 | 0.0000 | Up |
| COL10A1 | 1.2762 | 3.63E-16 | 1.66E-11 | 2.293 | 2.04E-10 | 0.0000 | Up |
| COL11A1 | 1.3072 | 4.98E-19 | 2.31E-14 | 2.283 | 5.75E-14 | 0.0000 | Up |
| COL12A1 | 1.1843 | 3.19E-16 | 1.46E-11 | 1.362 | 1.68E-12 | 0.0000 | Up |
| COL1A1 | 1.2781 | 9.21E-24 | 4.34E-19 | 1.258 | 1.88E-14 | 0.0000 | Up |
| COL1A2 | 1.1211 | 1.23E-13 | 5.53E-09 | 1.294 | 7.39E-14 | 0.0000 | Up |
| COL3A1 | 1.0686 | 4.08E-09 | 0.000172 | 1.173 | 5.92E-11 | 0.0000 | Up |
| COL4A1 | 1.1212 | 1.50E-17 | 6.90E-13 | 1.144 | 6.28E-09 | 0.0001 | Up |
| COL5A1 | 1.0987 | 1.39E-10 | 5.99E-06 | 1.178 | 8.08E-10 | 0.0000 | Up |
| COL5A2 | 1.1479 | 1.36E-16 | 6.24E-12 | 1.343 | 5.02E-11 | 0.0000 | Up |
| COL7A1 | 1.1714 | 2.17E-15 | 9.85E-11 | 1.163 | 4.44E-07 | 0.0077 | Up |
| COLGALT1 | 1.0619 | 6.04E-17 | 2.77E-12 | 1.066 | 6.48E-08 | 0.0011 | Up |
| COMP | 1.2068 | 1.11E-10 | 4.81E-06 | 1.500 | 9.69E-07 | 0.0166 | Up |
| COMT | 1.0707 | 2.36E-21 | 1.10E-16 | 1.056 | 9.87E-08 | 0.0017 | Up |
| COPS8 | 1.0555 | 9.54E-15 | 4.32E-10 | 1.117 | 6.01E-11 | 0.0000 | Up |
| CPNE1 | 1.1189 | 5.99E-23 | 2.81E-18 | 1.121 | 7.88E-10 | 0.0000 | Up |
| CPSF3 | 1.0632 | 3.38E-25 | 1.60E-20 | 1.150 | 1.75E-06 | 0.0298 | Up |

**Supplementary table 3.** Top 10% enriched GO terms (n=80) of the overlapped DEGs.

| ID | Description | GeneRatio | BgRatio | p.adjust | Count |
| --- | --- | --- | --- | --- | --- |
| GO:0042254 | ribosome biogenesis | 76/1731 | 297/18670 | 6.98E-13 | 76 |
| GO:0016072 | rRNA metabolic process | 65/1731 | 253/18670 | 4.70E-11 | 65 |
| GO:0006364 | rRNA processing | 58/1731 | 214/18670 | 6.71E-11 | 58 |
| GO:0006403 | RNA localization | 57/1731 | 230/18670 | 4.87E-09 | 57 |
| GO:0070482 | response to oxygen levels | 81/1731 | 394/18670 | 5.68E-09 | 81 |
| GO:0030198 | extracellular matrix organization | 76/1731 | 368/18670 | 1.72E-08 | 76 |
| GO:0062012 | regulation of small molecule metabolic process | 88/1731 | 459/18670 | 2.54E-08 | 88 |
| GO:0043062 | extracellular structure organization | 82/1731 | 422/18670 | 5.29E-08 | 82 |
| GO:0001666 | response to hypoxia | 73/1731 | 359/18670 | 6.13E-08 | 73 |
| GO:0016053 | organic acid biosynthetic process | 87/1731 | 463/18670 | 6.80E-08 | 87 |
| GO:0036293 | response to decreased oxygen levels | 74/1731 | 370/18670 | 8.33E-08 | 74 |
| GO:0071456 | cellular response to hypoxia | 50/1731 | 207/18670 | 9.22E-08 | 50 |
| GO:0071453 | cellular response to oxygen levels | 54/1731 | 234/18670 | 1.01E-07 | 54 |
| GO:0046394 | carboxylic acid biosynthetic process | 86/1731 | 462/18670 | 1.02E-07 | 86 |
| GO:0036294 | cellular response to decreased oxygen levels | 51/1731 | 217/18670 | 1.39E-07 | 51 |
| GO:0010565 | regulation of cellular ketone metabolic process | 45/1731 | 181/18670 | 2.05E-07 | 45 |
| GO:0006520 | cellular amino acid metabolic process | 72/1731 | 366/18670 | 2.05E-07 | 72 |
| GO:0000086 | G2/M transition of mitotic cell cycle | 55/1731 | 247/18670 | 2.05E-07 | 55 |
| GO:0034470 | ncRNA processing | 74/1731 | 384/18670 | 2.79E-07 | 74 |
| GO:0072522 | purine-containing compound biosynthetic process | 64/1731 | 313/18670 | 3.14E-07 | 64 |
| GO:0009165 | nucleotide biosynthetic process | 74/1731 | 386/18670 | 3.21E-07 | 74 |
| GO:0006164 | purine nucleotide biosynthetic process | 62/1731 | 300/18670 | 3.35E-07 | 62 |
| GO:0006260 | DNA replication | 58/1731 | 274/18670 | 4.09E-07 | 58 |
| GO:2000278 | regulation of DNA biosynthetic process | 32/1731 | 108/18670 | 4.09E-07 | 32 |
| GO:0006401 | RNA catabolic process | 75/1731 | 397/18670 | 4.13E-07 | 75 |
| GO:1901293 | nucleoside phosphate biosynthetic process | 74/1731 | 390/18670 | 4.17E-07 | 74 |
| GO:1904816 | positive regulation of protein localization to chromosome, telomeric region | 10/1731 | 12/18670 | 5.65E-07 | 10 |
| GO:2000573 | positive regulation of DNA biosynthetic process | 24/1731 | 67/18670 | 5.68E-07 | 24 |
| GO:0044839 | cell cycle G2/M phase transition | 56/1731 | 266/18670 | 7.69E-07 | 56 |
| GO:0006405 | RNA export from nucleus | 36/1731 | 135/18670 | 7.69E-07 | 36 |
| GO:0001503 | ossification | 74/1731 | 398/18670 | 8.78E-07 | 74 |
| GO:0051168 | nuclear export | 45/1731 | 194/18670 | 1.08E-06 | 45 |
| GO:0006402 | mRNA catabolic process | 69/1731 | 364/18670 | 1.21E-06 | 69 |
| GO:0140014 | mitotic nuclear division | 55/1731 | 264/18670 | 1.36E-06 | 55 |
| GO:0006732 | coenzyme metabolic process | 74/1731 | 403/18670 | 1.36E-06 | 74 |
| GO:0046390 | ribose phosphate biosynthetic process | 60/1731 | 300/18670 | 1.38E-06 | 60 |
| GO:0009260 | ribonucleotide biosynthetic process | 59/1731 | 293/18670 | 1.38E-06 | 59 |
| GO:0009152 | purine ribonucleotide biosynthetic process | 57/1731 | 280/18670 | 1.59E-06 | 57 |
| GO:0051052 | regulation of DNA metabolic process | 77/1731 | 429/18670 | 1.71E-06 | 77 |
| GO:0006611 | protein export from nucleus | 42/1731 | 179/18670 | 1.94E-06 | 42 |
| GO:0048285 | organelle fission | 79/1731 | 449/18670 | 2.54E-06 | 79 |
| GO:0051054 | positive regulation of DNA metabolic process | 49/1731 | 228/18670 | 2.54E-06 | 49 |
| GO:0007059 | chromosome segregation | 62/1731 | 321/18670 | 2.61E-06 | 62 |
| GO:0000819 | sister chromatid segregation | 43/1731 | 189/18670 | 3.13E-06 | 43 |
| GO:0071897 | DNA biosynthetic process | 44/1731 | 196/18670 | 3.23E-06 | 44 |
| GO:0051169 | nuclear transport | 65/1731 | 346/18670 | 3.40E-06 | 65 |
| GO:0000280 | nuclear division | 73/1731 | 407/18670 | 3.51E-06 | 73 |
| GO:0072331 | signal transduction by p53 class mediator | 54/1731 | 267/18670 | 3.77E-06 | 54 |
| GO:1901988 | negative regulation of cell cycle phase transition | 54/1731 | 267/18670 | 3.77E-06 | 54 |
| GO:1904814 | regulation of protein localization to chromosome, telomeric region | 10/1731 | 14/18670 | 3.88E-06 | 10 |
| GO:0031145 | anaphase-promoting complex-dependent catabolic process | 25/1731 | 81/18670 | 4.67E-06 | 25 |
| GO:1901991 | negative regulation of mitotic cell cycle phase transition | 51/1731 | 248/18670 | 4.86E-06 | 51 |
| GO:1904358 | positive regulation of telomere maintenance via telomere lengthening | 16/1731 | 37/18670 | 6.08E-06 | 16 |
| GO:0060249 | anatomical structure homeostasis | 76/1731 | 437/18670 | 6.08E-06 | 76 |
| GO:0000723 | telomere maintenance | 38/1731 | 162/18670 | 6.89E-06 | 38 |
| GO:0010389 | regulation of G2/M transition of mitotic cell cycle | 43/1731 | 196/18670 | 7.53E-06 | 43 |
| GO:1904874 | positive regulation of telomerase RNA localization to Cajal body | 10/1731 | 15/18670 | 9.31E-06 | 10 |
| GO:0000070 | mitotic sister chromatid segregation | 36/1731 | 151/18670 | 9.31E-06 | 36 |
| GO:0006913 | nucleocytoplasmic transport | 63/1731 | 343/18670 | 1.02E-05 | 63 |
| GO:1901990 | regulation of mitotic cell cycle phase transition | 76/1731 | 444/18670 | 1.07E-05 | 76 |
| GO:0071426 | ribonucleoprotein complex export from nucleus | 32/1731 | 127/18670 | 1.16E-05 | 32 |
| GO:0045930 | negative regulation of mitotic cell cycle | 62/1731 | 338/18670 | 1.29E-05 | 62 |
| GO:0010948 | negative regulation of cell cycle process | 65/1731 | 361/18670 | 1.29E-05 | 65 |
| GO:0048608 | reproductive structure development | 74/1731 | 431/18670 | 1.30E-05 | 74 |
| GO:0071166 | ribonucleoprotein complex localization | 32/1731 | 128/18670 | 1.33E-05 | 32 |
| GO:0071496 | cellular response to external stimulus | 62/1731 | 339/18670 | 1.35E-05 | 62 |
| GO:0061458 | reproductive system development | 74/1731 | 434/18670 | 1.66E-05 | 74 |
| GO:1903405 | protein localization to nuclear body | 8/1731 | 10/18670 | 1.75E-05 | 8 |
| GO:1904851 | positive regulation of establishment of protein localization to telomere | 8/1731 | 10/18670 | 1.75E-05 | 8 |
| GO:1904867 | protein localization to Cajal body | 8/1731 | 10/18670 | 1.75E-05 | 8 |
| GO:0070200 | establishment of protein localization to telomere | 10/1731 | 16/18670 | 1.84E-05 | 10 |
| GO:0072330 | monocarboxylic acid biosynthetic process | 62/1731 | 343/18670 | 1.89E-05 | 62 |
| GO:0009108 | coenzyme biosynthetic process | 51/1731 | 261/18670 | 1.89E-05 | 51 |
| GO:1902850 | microtubule cytoskeleton organization involved in mitosis | 32/1731 | 131/18670 | 2.05E-05 | 32 |
| GO:0051973 | positive regulation of telomerase activity | 15/1731 | 36/18670 | 2.05E-05 | 15 |
| GO:0042180 | cellular ketone metabolic process | 49/1731 | 248/18670 | 2.20E-05 | 49 |
| GO:1902749 | regulation of cell cycle G2/M phase transition | 44/1731 | 213/18670 | 2.36E-05 | 44 |
| GO:0061013 | regulation of mRNA catabolic process | 42/1731 | 199/18670 | 2.36E-05 | 42 |
| GO:1901987 | regulation of cell cycle phase transition | 79/1731 | 480/18670 | 2.45E-05 | 79 |
| GO:0050657 | nucleic acid transport | 41/1731 | 193/18670 | 2.61E-05 | 41 |

**Supplementary table 4.** Enriched KEGG pathways of the overlapped DEGs.

| ID | Description | GeneRatio | BgRatio | p.adjust | Count |
| --- | --- | --- | --- | --- | --- |
| hsa03030 | DNA replication | 16/569 | 36/3815 | 0.0022 | 16 |
| hsa03050 | Proteasome | 16/569 | 46/3815 | 0.0342 | 16 |
| hsa03430 | Mismatch repair | 10/569 | 23/3815 | 0.0342 | 10 |
| hsa00920 | Sulfur metabolism | 6/569 | 10/3815 | 0.0369 | 6 |
|  |  |  |  |  |  |

**Supplementary table 5.** Top 20 enrichments of GSEA in KC2 sub-group.

| KEGG pathway | SIZE | ES | NES | NOM p-val | FDR q-val |
| --- | --- | --- | --- | --- | --- |
| KEGG_FOCAL_ADHESION | 167 | 0.6865 | 2.3156 | 0.0000 | 0.0000 |
| KEGG_VASCULAR_SMOOTH_MUSCLE_CONTRACTION | 82 | 0.6201 | 2.2435 | 0.0000 | 0.0000 |
| KEGG_LEUKOCYTE_TRANSENDOTHELIAL_MIGRATION | 95 | 0.6288 | 2.1990 | 0.0000 | 0.0005 |
| KEGG_DILATED_CARDIOMYOPATHY | 60 | 0.6701 | 2.1597 | 0.0000 | 0.0010 |
| KEGG_REGULATION_OF_ACTIN_CYTOSKELETON | 170 | 0.5647 | 2.1422 | 0.0000 | 0.0011 |
| KEGG_CELL_ADHESION_MOLECULES_CAMS | 104 | 0.6966 | 2.1374 | 0.0000 | 0.0010 |
| KEGG_HYPERTROPHIC_CARDIOMYOPATHY_HCM | 57 | 0.6321 | 2.1243 | 0.0021 | 0.0014 |
| KEGG_ECM_RECEPTOR_INTERACTION | 64 | 0.7791 | 2.1050 | 0.0000 | 0.0019 |
| KEGG_MELANOGENESIS | 76 | 0.5480 | 2.0866 | 0.0000 | 0.0021 |
| KEGG_AXON_GUIDANCE | 104 | 0.5511 | 2.0758 | 0.0000 | 0.0022 |
| KEGG_GLYCOSAMINOGLYCAN_BIOSYNTHESIS_CHONDROITIN_SULFATE | 20 | 0.8094 | 2.0632 | 0.0000 | 0.0026 |
| KEGG_NEUROACTIVE_LIGAND_RECEPTOR_INTERACTION | 89 | 0.5642 | 2.0413 | 0.0000 | 0.0036 |
| KEGG_VIRAL_MYOCARDITIS | 56 | 0.6543 | 2.0184 | 0.0020 | 0.0051 |
| KEGG_TGF_BETA_SIGNALING_PATHWAY | 75 | 0.5514 | 2.0047 | 0.0020 | 0.0054 |
| KEGG_MELANOMA | 51 | 0.5472 | 1.9942 | 0.0000 | 0.0062 |
| KEGG_BASAL_CELL_CARCINOMA | 41 | 0.6118 | 1.9861 | 0.0021 | 0.0066 |
| KEGG_JAK_STAT_SIGNALING_PATHWAY | 100 | 0.5832 | 1.9833 | 0.0000 | 0.0063 |
| KEGG_COMPLEMENT_AND_COAGULATION_CASCADES | 48 | 0.6690 | 1.9822 | 0.0046 | 0.0061 |
| KEGG_FC_GAMMA_R_MEDIATED_PHAGOCYTOSIS | 85 | 0.5477 | 1.9809 | 0.0000 | 0.0058 |
